# Supplementary material for: High PANX1 Expression Leads to Neutrophil Recruitment and the Formation of a High Adenosine Immunosuppressive Tumor Microenvironment in Basal-like Breast Cancer
Source: Cancers (Basel). 2022 Jul 11;14(14):3369. doi: 10.3390/cancers14143369 (PMC9323990; doi:10.3390/cancers14143369)
Supplement: Supplementary file 1 [file cancers-14-03369-s001.zip › AF42 Informed-consent-statement.pdf]

AF42 浙江大学医学院附属第二医院人体研究知情同意书

尊敬的患者：

我们邀请您参加一项“三阴乳腺癌高表达 PANX1 通过 ATP 极化 N2 型中性粒细胞形成高嘌呤核苷免疫抑制微环境的机制研究”（方案编号：审研 2020-337）的临床研究，在您决定是否参加这项研究之前，请仔细阅读以下内容，它可以帮助您了解该项研究以及为什么要进行这项研究，研究的程序和期限，参加研究后可能给您带来的益处、风险和不便。

您参加本项研究是自愿的。本次研究已通过本研究机构伦理审查委员会审查。

以下是本项研究的介绍：

一、研究背景和研究目的

乳腺癌是女性最常见的恶性肿瘤之一，其中三阴乳腺癌（Triple negative breast cancer，TNBC）不表达雌激素受体（ER）、孕激素受体（PR）及人类表皮生长因子受体 2（HER2），占乳腺癌总体的 15-20％，具有侵袭强、易复发转移的特点，是乳腺癌中预后较差的亚群，且缺乏特异性治疗手段。尽管乳腺癌总体免疫原性不强，但较之其他乳腺癌亚型，TNBC 具有较高的免疫原性，表现为具有高肿瘤突变负荷和高免疫细胞浸润。对于 TNBC 的免疫治疗，尽管有部分临床试验取得了一定疗效，但也有一些临床试验以阴性结果而告终，同时带来较多的药物副作用，提示需深入探索 TNBC 和肿瘤免疫微环境的关系，寻找关键免疫细胞及靶基因，具有重要的临床和科学意义。

二、具体程序和流程

1．获取患者病理组织石蜡块；2．获取患者新鲜手术切除组织；3．将患者临床资料进行整合分析。研究期限为 2020 年 3 月—2024 年 1 月，按具体疾病常规随访复查即可。

三、如果参加研究您需要做什么

如果您同意参与这项研究，我们将对您进行编号，建立病历档案。由于临床诊断或治疗需要，您要进行某些外科手术，手术中切除的组织除供临床常规病理检查之外可能还有一些剩余将丢弃，我们将收集这些剩余的组织标本进行研究。同时采集疾病有关的信息资料，包括发病过程、家族史、以前就诊情况及曾经做过一些检验、检查结果等。

四、参加本研究可能给您带来的受益

通过对您的标本和信息资料进行研究，将有助于对疾病作出诊断，为您的治疗提供必要的建议，或为疾病的研究提供有益的信息。

五、参加本研究可能发生的不良反应、风险以及风险防范措施

对于您来说，所有的信息将是保密的。您的手术和诊疗将由专业医师操作，我们仅采集一些临床病理检查剩余的组织标本和疾病有关的信息资料。本研究不涉及患者诊断和治疗干预，无明显风险和不良反应。

六、费用情况说明

本研究不会增加患者额外的费用。

七、参与研究的补偿，包括损伤的赔偿

如果您因参与这项研究而受到伤害：如发生与临床研究相关的损害时，您可以获得免费治疗和 / 或相应的补偿。

八、替代方案

若您不愿意参加本研究，不存在替代方案，不会影响您的常规临床治疗。

九、您个人信息的保密

您的医疗记录（包括研究病历及理化检查报告等）将按规定保存在医院。除研究者、伦理委员会、监查、稽查、药政管理部门等相关人员将被允许查阅您的医疗记录外，其他与研究无关的人员在未得到允许的情况下，无权查阅您的医疗记录。本研究结果的公开报告将不会披露您的个人身份。我们将在允许的范围内，尽一切努力保护您个人医疗资料的隐私。

十、终止参加研究

是否参加本项研究完全取决于您的自愿。您可以拒绝参加此项研究，或在研究过程中的任何时间无理由退出研究，这都不会影响您和医生的关系，都不会影响对您的医疗或有其他方面利益的损失。

十一、伦理委员会

本研究已向浙江大学医学院附属第二医院人体研究伦理委员会报告，经委员会的全面审查和包括对受试者的风险评估，并获得了批准。在研究中过程中，**有关伦理和权益事宜可联系浙江大学医学院附属第二医院人体研究伦理委员会，电话：白天 0571-87783759； 晚上（总值班）：13757118366；邮箱地址： HREC2013@126.com**

我确认已阅读并理解了本研究的知情同意书，自愿接受本研究中的治疗方法，并同意将我的医疗数据用于本研究的发表。

受试者签名：__________ 联系方式：______________ 日期：______________

代理人签名：______________与受试者关系______________联系方式_____________ 日期（如果需要）

见证人（如果需要）：______________联系方式：______________ 日期：______________

我确认已向患者解释了本研究的详细情况，包括其权利以及可能的受益和风险，并给其一份签署过的知情同意书副本。

研究者签名： 陈武臻

联系方式： 13067847087（手机） 日期： 2020-2-15
