# Supplementary material for: High PANX1 Expression Leads to Neutrophil Recruitment and the Formation of a High Adenosine Immunosuppressive Tumor Microenvironment in Basal-like Breast Cancer
Source: Cancers (Basel). 2022 Jul 11;14(14):3369. doi: 10.3390/cancers14143369 (PMC9323990; doi:10.3390/cancers14143369)
Supplement: Supplementary file 1 [file cancers-14-03369-s001.zip › cancers-1790081 - Supplementary Fig.pptx]

## Slide 1
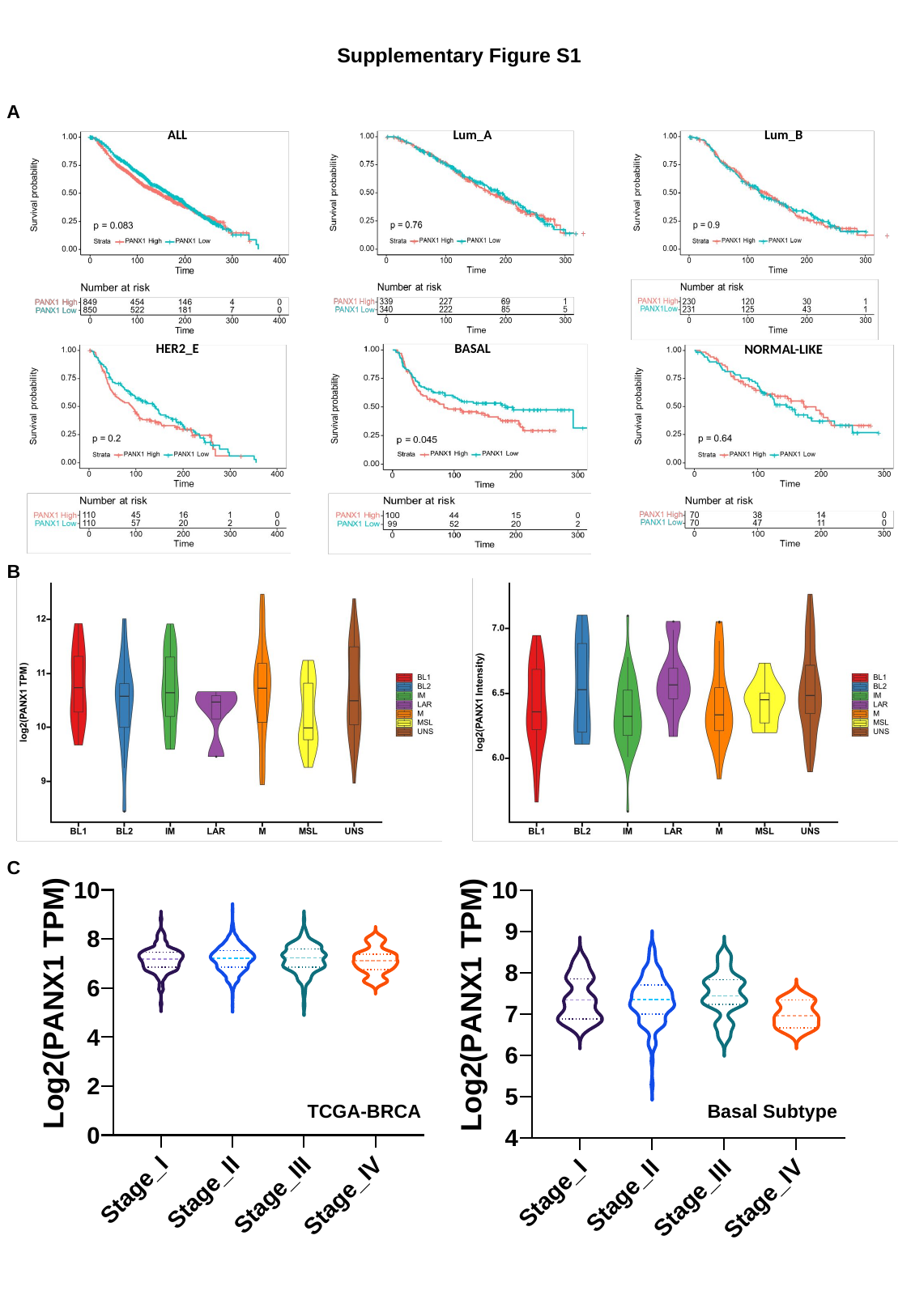

Supplementary Figure S1
A
ALL
Lum_A
Lum_B
HER2_E
BASAL
NORMAL-LIKE
B
C
TCGA-BRCA
Basal Subtype

## Slide 2
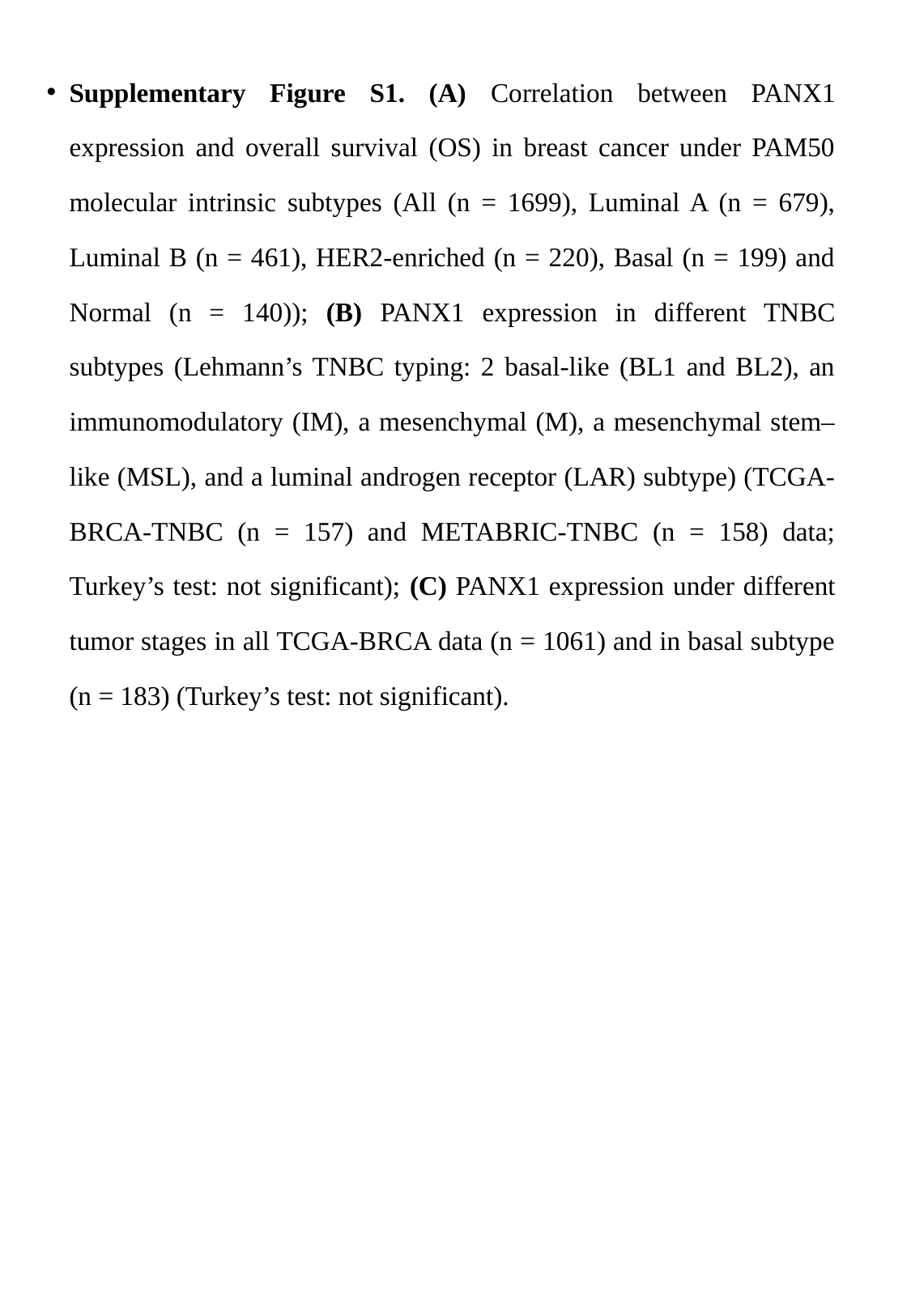

Supplementary Figure S1. (A) Correlation between PANX1 expression and overall survival (OS) in breast cancer under PAM50 molecular intrinsic subtypes (All (n = 1699), Luminal A (n = 679), Luminal B (n = 461), HER2-enriched (n = 220), Basal (n = 199) and Normal (n = 140)); (B) PANX1 expression in different TNBC subtypes (Lehmann’s TNBC typing: 2 basal-like (BL1 and BL2), an immunomodulatory (IM), a mesenchymal (M), a mesenchymal stem–like (MSL), and a luminal androgen receptor (LAR) subtype) (TCGA-BRCA-TNBC (n = 157) and METABRIC-TNBC (n = 158) data; Turkey’s test: not significant); (C) PANX1 expression under different tumor stages in all TCGA-BRCA data (n = 1061) and in basal subtype (n = 183) (Turkey’s test: not significant).

## Slide 3
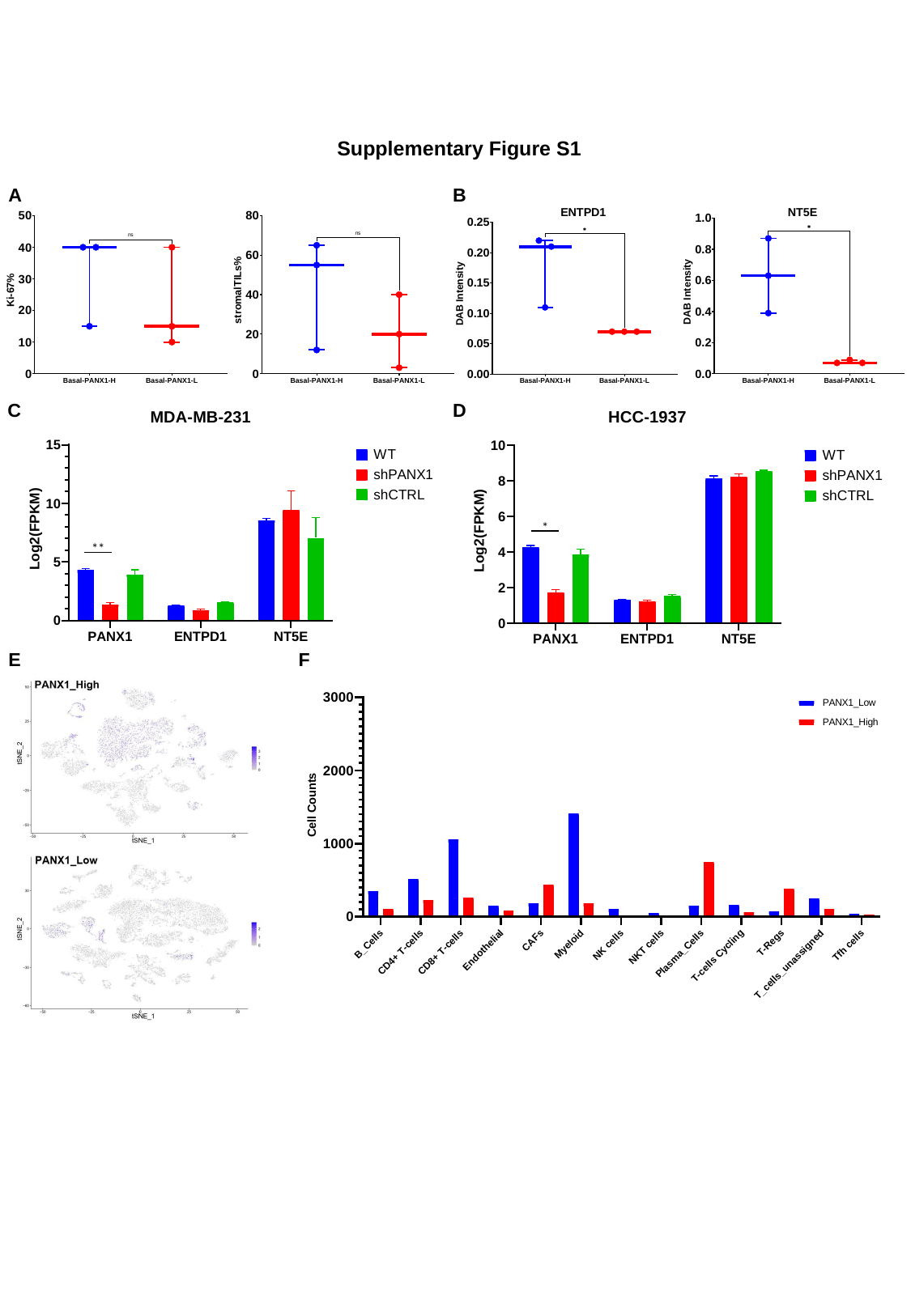

Supplementary Figure S1
A
B
ENTPD1
NT5E
C
D
*
**
E
F

## Slide 4
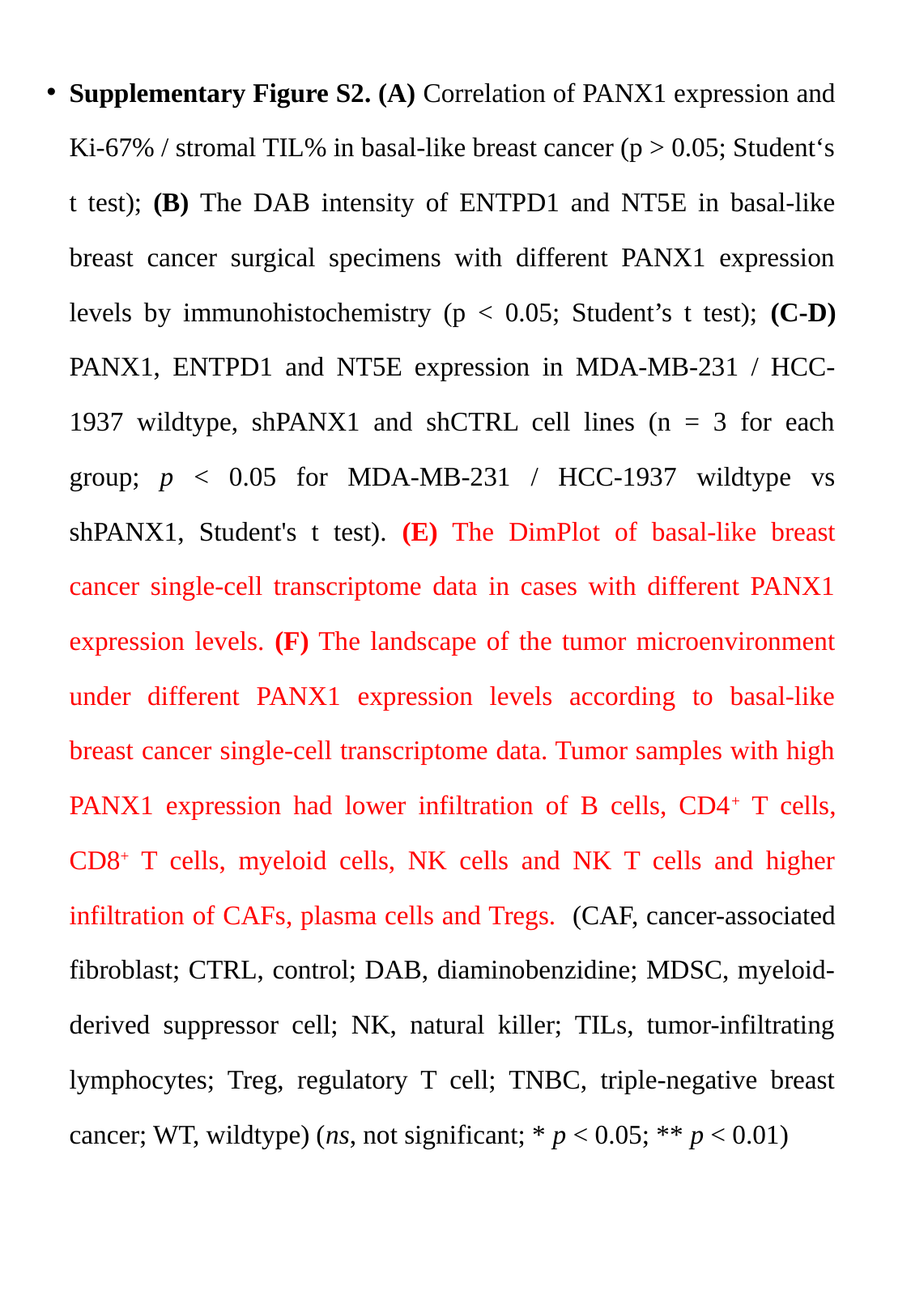

Supplementary Figure S2. (A) Correlation of PANX1 expression and Ki-67% / stromal TIL% in basal-like breast cancer (p > 0.05; Student‘s t test); (B) The DAB intensity of ENTPD1 and NT5E in basal-like breast cancer surgical specimens with different PANX1 expression levels by immunohistochemistry (p < 0.05; Student’s t test); (C-D) PANX1, ENTPD1 and NT5E expression in MDA-MB-231 / HCC-1937 wildtype, shPANX1 and shCTRL cell lines (n = 3 for each group; p < 0.05 for MDA-MB-231 / HCC-1937 wildtype vs shPANX1, Student's t test). (E) The DimPlot of basal-like breast cancer single-cell transcriptome data in cases with different PANX1 expression levels. (F) The landscape of the tumor microenvironment under different PANX1 expression levels according to basal-like breast cancer single-cell transcriptome data. Tumor samples with high PANX1 expression had lower infiltration of B cells, CD4+ T cells, CD8+ T cells, myeloid cells, NK cells and NK T cells and higher infiltration of CAFs, plasma cells and Tregs. (CAF, cancer-associated fibroblast; CTRL, control; DAB, diaminobenzidine; MDSC, myeloid-derived suppressor cell; NK, natural killer; TILs, tumor-infiltrating lymphocytes; Treg, regulatory T cell; TNBC, triple-negative breast cancer; WT, wildtype) (ns, not significant; * p < 0.05; ** p < 0.01)
