# Supplementary material for: High PANX1 Expression Leads to Neutrophil Recruitment and the Formation of a High Adenosine Immunosuppressive Tumor Microenvironment in Basal-like Breast Cancer
Source: Cancers (Basel). 2022 Jul 11;14(14):3369. doi: 10.3390/cancers14143369 (PMC9323990; doi:10.3390/cancers14143369)
Supplement: Supplementary file 1 [file cancers-14-03369-s001.zip › Ethical approval.pdf]

科研项目审查通知函

IRB Action Letter

尊敬的 陈武臻 研究者：

您提交的编号为 审研2020-337 研究方案 三阴乳腺癌高表达PANX1通过ATP极化衰老中性粒细胞形成高嘌呤核苷免疫抑制微环境的机制研究 经浙江大学医学院附属第二医院人体研究伦理审查。审查结果为 同意 。[illegible]

主任/副主任委员签名 吴志英

2020 年 03 月 12 日

浙江大学医学院附属第二医院人体研究伦理委员会
